# Supplementary material for: Bioinspired Pd-Cu Alloy Nanoparticles as Accept Agent for Dye Degradation Performances
Source: Int J Mol Sci. 2022 Nov 15;23(22):14072. doi: 10.3390/ijms232214072 (PMC9698934; doi:10.3390/ijms232214072)
Supplement: Supplementary file 1 [file ijms-23-14072-s001.zip › ijms-1979714-supplementary.pdf]

Supporting Information

Bioinspired Pd-Cu Alloy Nanoparticles as Accept Agent for Dye Degradation Performances

Shiyue Chen [1,2,†], Yujun Yang [3,†], Mingjun Zhang [1,2], Xiaohong Ma [4], Xiaoxiao He [1,2], Teng Wang [1,2], Xi Hu [1,2] and Xiang Mao [1,2,*]

[1] State Key Laboratory of Ultrasound in Medicine and Engineering, College of Biomedical Engineering, Chongqing Medical University, Chongqing 400016, China
[2] Chongqing Key Laboratory of Biomedical Engineering, College of Biomedical Engineering, Chongqing Medical University, Chongqing 400016, China
[3] Chongqing Medical Laboratory Microfluidics and SPRi Engineering Research Center, Key Laboratory of Laboratory Medical Diagnostics, Ministry of Education, Department of Laboratory Medicine, Chongqing Medical University, Chongqing 400016, China
[4] State Key Laboratory of Multi-Phase Complex Systems, Institute of Process Engineering, Chinese Academy of Sciences, Beijing 100190, China
* Correspondence: maox@cqmu.edu.cn
† These authors contributed equally to this work.

Supporting Information

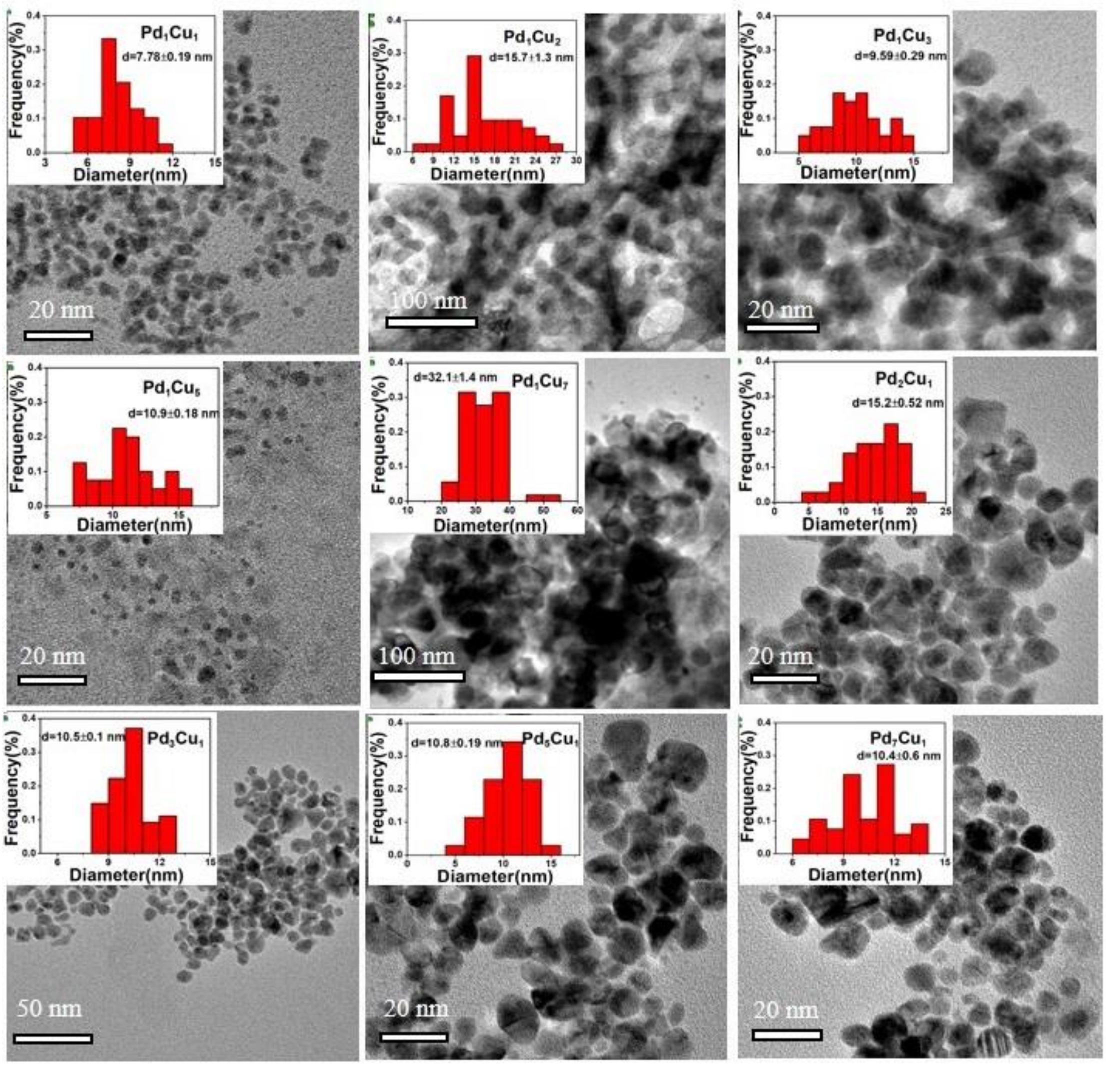

Figure. S1. TEM images and size distribution histogram of the Pd-Cu alloy NPs with different precursor ratios.

Supporting Information

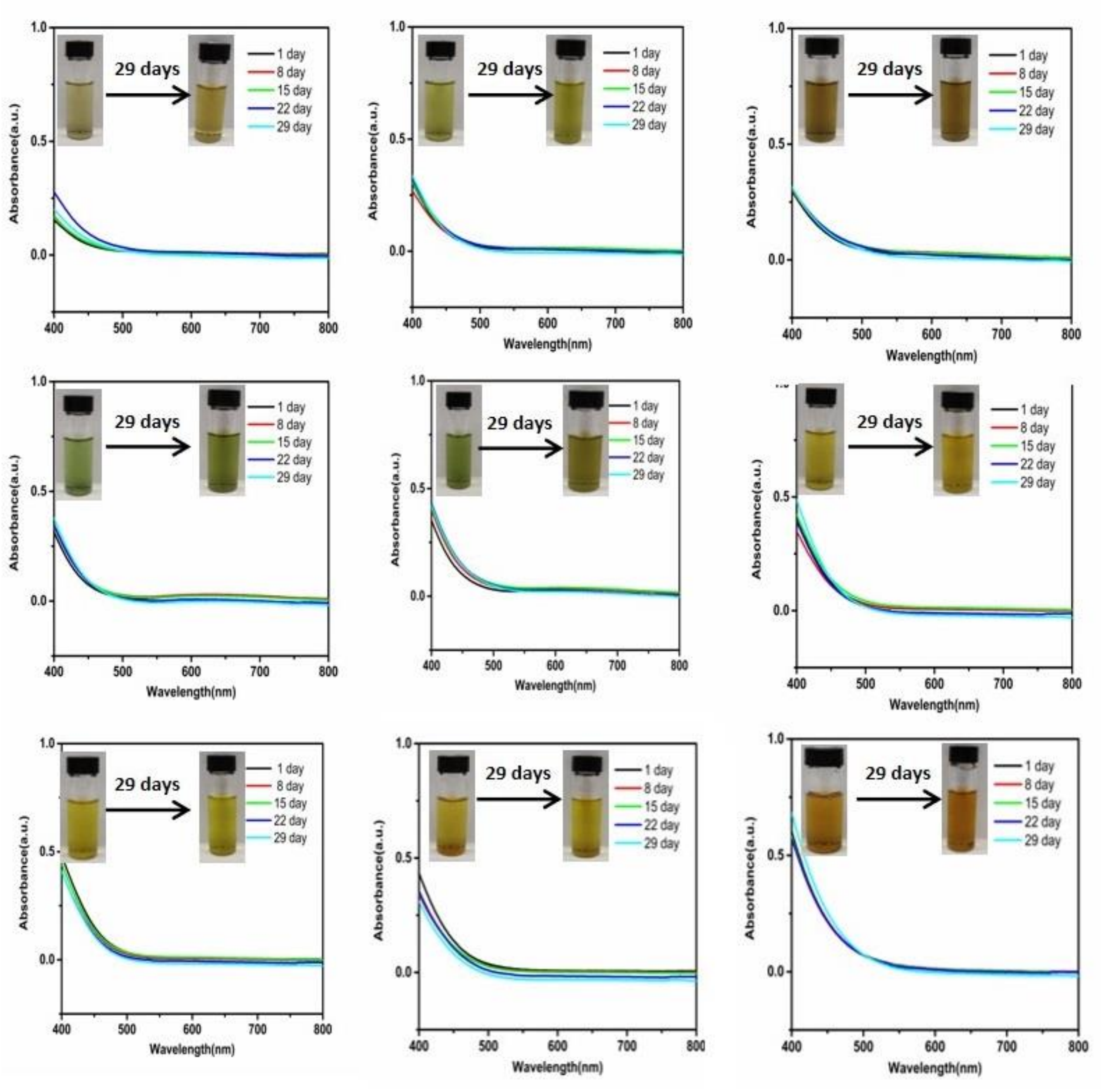

Figure. S2. UV-*vis* images and colloidal states of the Pd-Cu alloy NPs with different precursor ratios.

Supporting Information

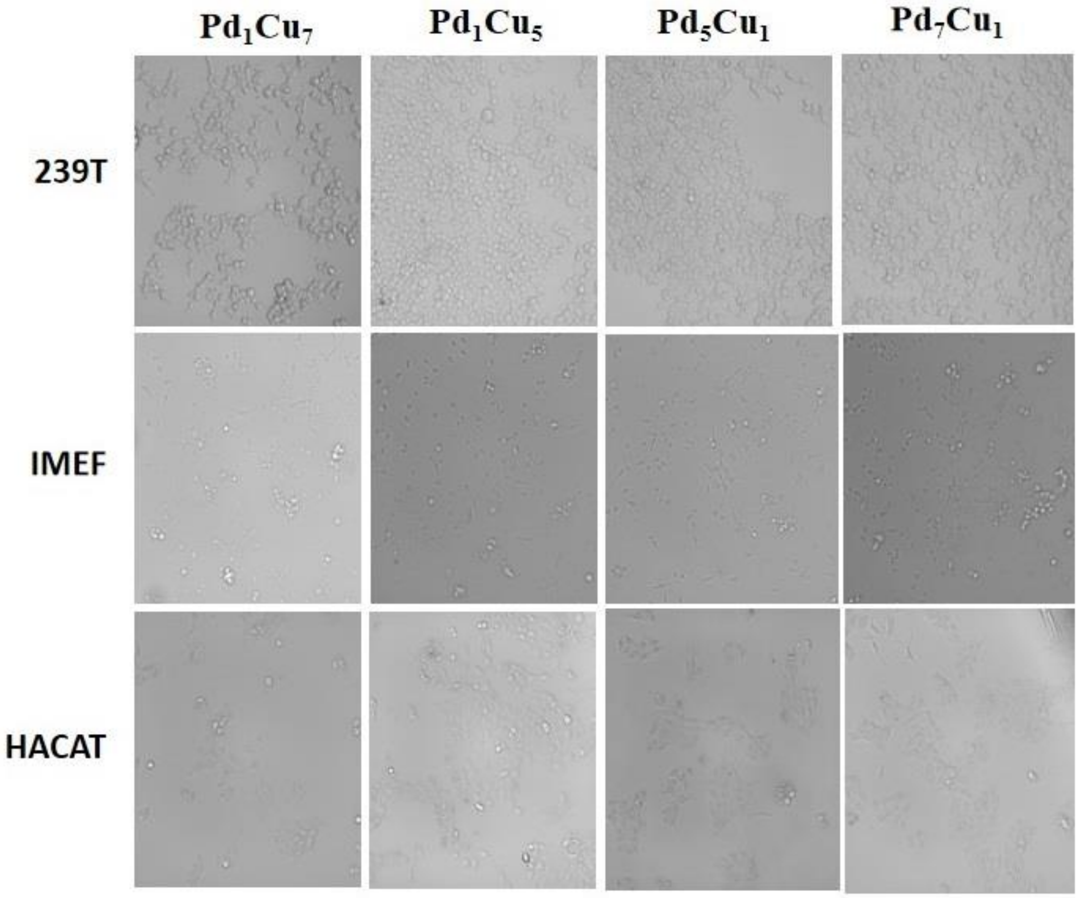

Figure. S3. Representative light microscopy images of 239T cell, IMEF cell, HACAT cell, which were treated by using 50 μg mL^{-1} colloidal solutions (Pd_1Cu_5, Pd_1Cu_7, Pd_5Cu_1, Pd_7Cu_1 alloy NPs).

Supporting Information

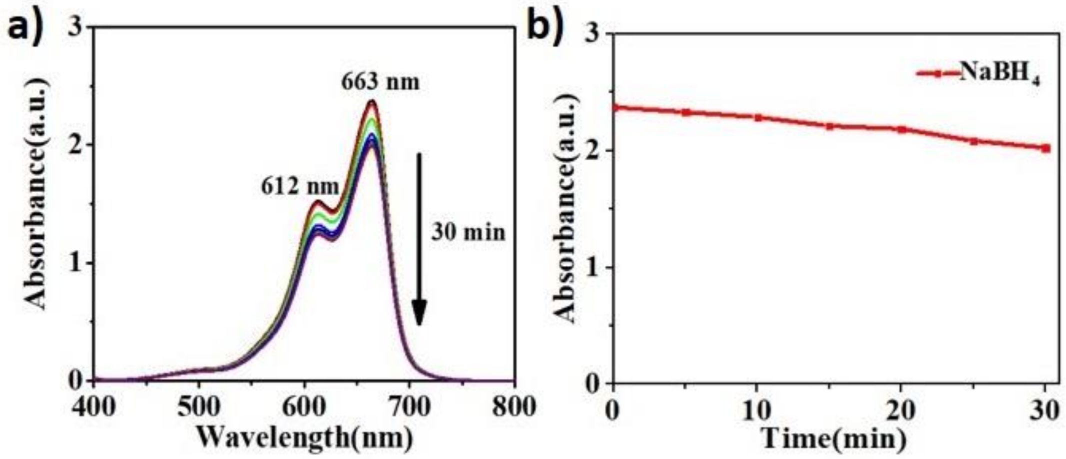

Figure. S4. a) Absorption spectra of MB in the presence of 0.1 mL of 0.2 M NaBH4. b) Absorption spectra recorded at 400 nm was affected by time.

Supporting Information

Table S1: The concentration of substrate MB was controlled at 0.07 mM, and the content of catalyst was controlled at 0.01 mg. The influence of different catalysts on the reaction rate was compared, and the result was represented by K_{app}, and the higher the value of K_{app}, the faster the reaction rate.

Samples	Reduction time(s)	Apparent rate constant (K_{app}, 10^{-3} s^{-1})	Activity parameter (K, s^{-1} g^{-1})
Pd_1Cu_1	270	12.9	1290
Pd_1Cu_2	240	14.6	1460
Pd_1Cu_3	210	17.1	1710
Pd_1Cu_5	180	20.0	2000
Pd_1Cu_7	150	24.0	2400
Pd_2Cu_1	90	41.1	4110
Pd_3Cu_1	60	66.7	6670
Pd_5Cu_1	40	75.0	7500
Pd_7Cu_1	30	100.0	10000

Supporting Information

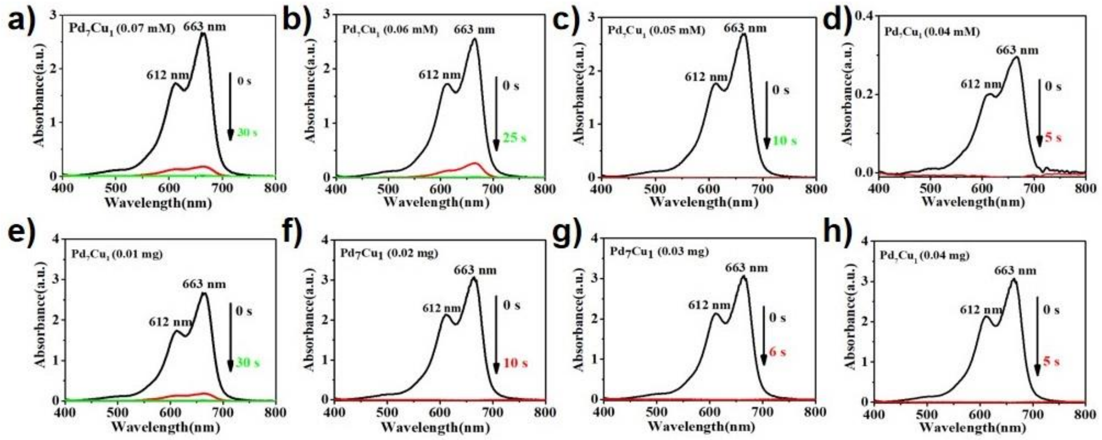

Figure. S5. UV-*vis* absorption spectra of degradation of a-d) with varying concentration of MB (0.04 - 0.07 mM) and constant amount (0.01 mg) of catalyst, e-h) with varying amount of catalyst (0.01 - 0.04 mg), and constant amount (0.07 mM) of MB dye.

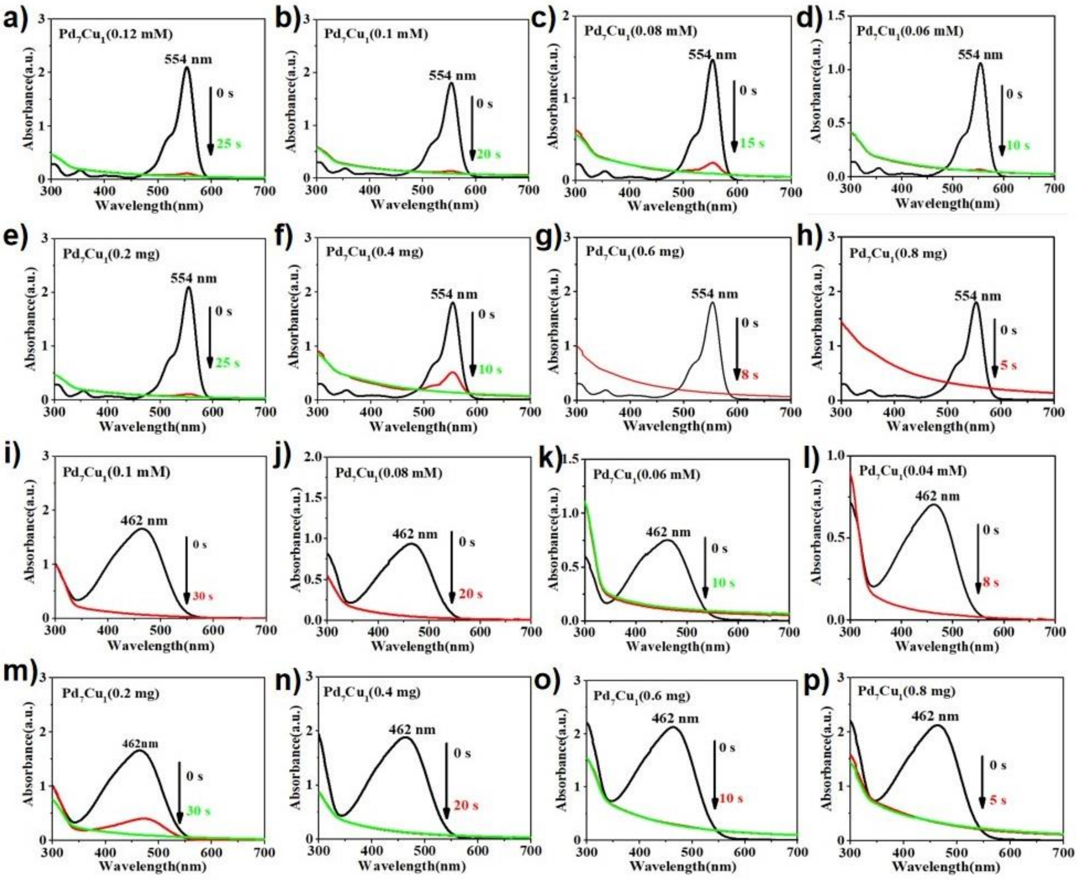

Figure. S6. UV-*vis* absorption spectra of degradation of a-d) with varying concentration of RhB (0.06 - 0.12 mM) and constant amount (0.2 mg) of catalyst, e-h) with varying amount of catalyst (0.2 - 0.8 mg), and constant amount (0.12 mM) of RhB dye, i - l) with varying concentration of MO (0.04 - 0.1 mM) and constant amount (0.2 mg) of catalyst, m - p) with varying amount of catalyst (0.2 - 0.8 mg), and constant amount (0.1 mM) of MO dye.

Supporting Information

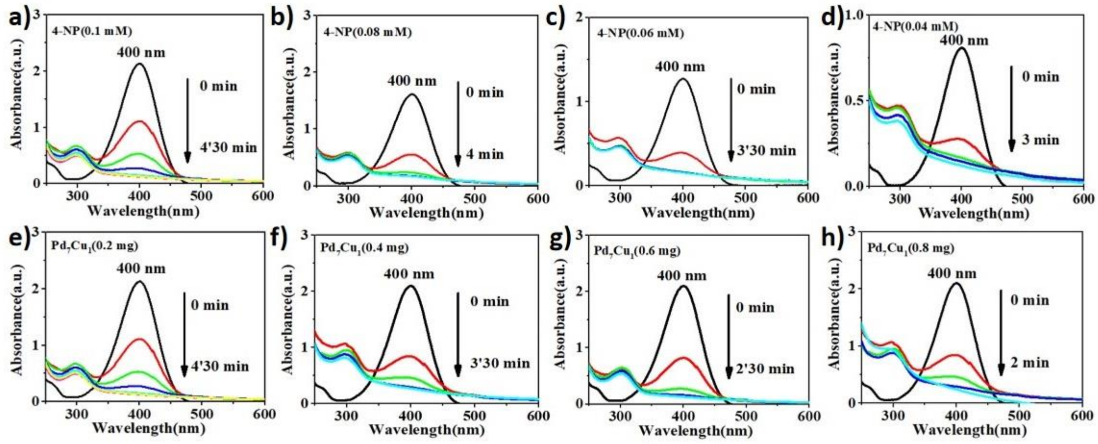

Figure. S7. UV-vis absorption spectra of degradation of a-d) with varying concentration of 4-NP (0.04 – 0.1 mM) and constant amount (0.2 mg) of catalyst, e-h) with varying amount of catalyst (0.2 - 0.8 mg), and constant amount (0.1 mM) of 4-NP.

Supporting Information

Table S2. Summary of Reaction Time (s), Apparent Rate Constant (Kapp) and Activity Parameter (K) for Degradation of 4-NP with Varying Concentration and Catalytic Loading.

concentration of 4-NP (mM)	amount of catalyst (mg)	reaction (s)	Apparent rate constant (Kapp, 10^{-3} s^{-1})	Activity parameter (K, s^{-1} g^{-1})
0.04	/0.2	180	11.2	56
0.06	0.2	210	9.9	49.5
0.08	0.2	240	8.7	43.5
0.1	0.2	270	6.9	34.5
0.1	0.4	210	8.5	21.25
0.1	0.6	150	19.1	31.83
0.1	0.8	120	26.2	32.75

Supporting Information

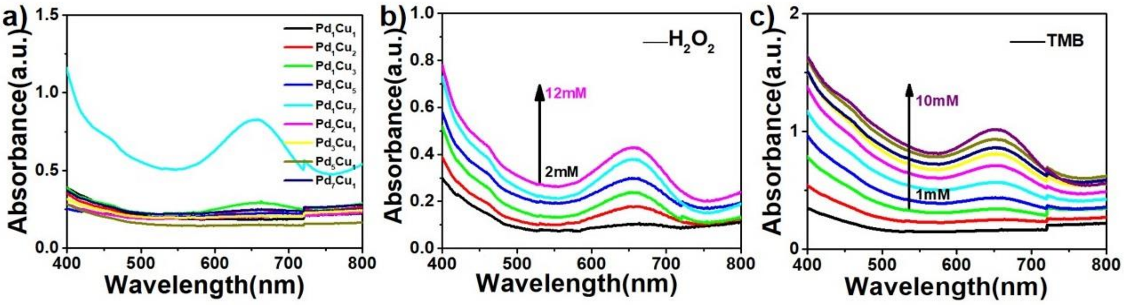

Figure. S8. a) UV-*vis* absorbance spectra of peroxidase-like activity in different concentrations of the Pd-Cu alloy NPs with different precursor ratios; b) UV-*vis* absorbance spectra of peroxidase-like activity in different concentrations of H_2O_2; c) UV-*vis* absorbance spectra of peroxidase-like activity in different concentrations of TMB.

Supporting Information

Table S3: Kinetic parameters of Pd_7Cu_1 alloy NPs.

Enzyme	Substrate	K_m (mM)	V_{max} (mM s^{-1})
Pd_1Cu_7 alloy NPs	H_2O_2	18.26	4.34
Pd_1Cu_7 alloy NPs	TMB	10.62	7.69

Supporting Information

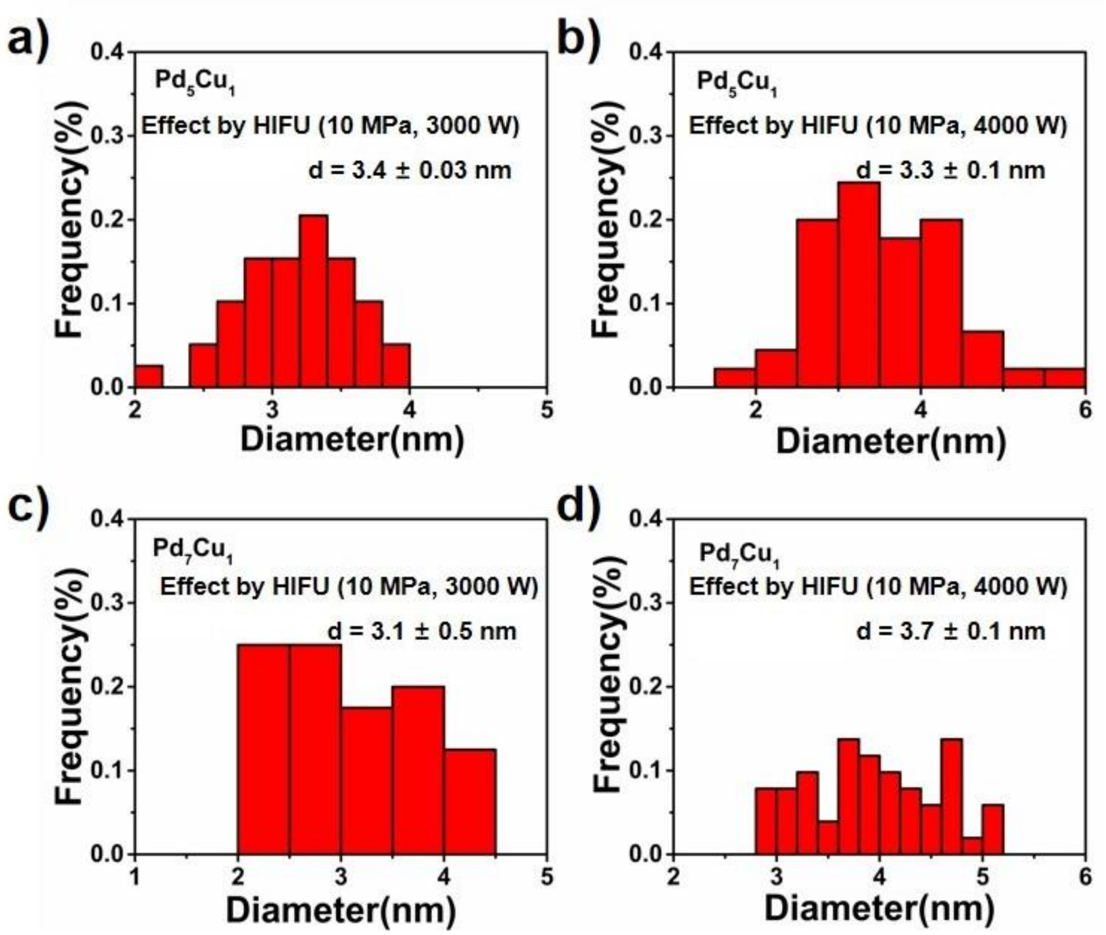

Figure. S9. Size distribution histogram of Pd_5Cu_1 alloy NPs and Pd_7Cu_1 alloy NPs effected by HIFU with different ultrasonic parameters.

Supporting Information

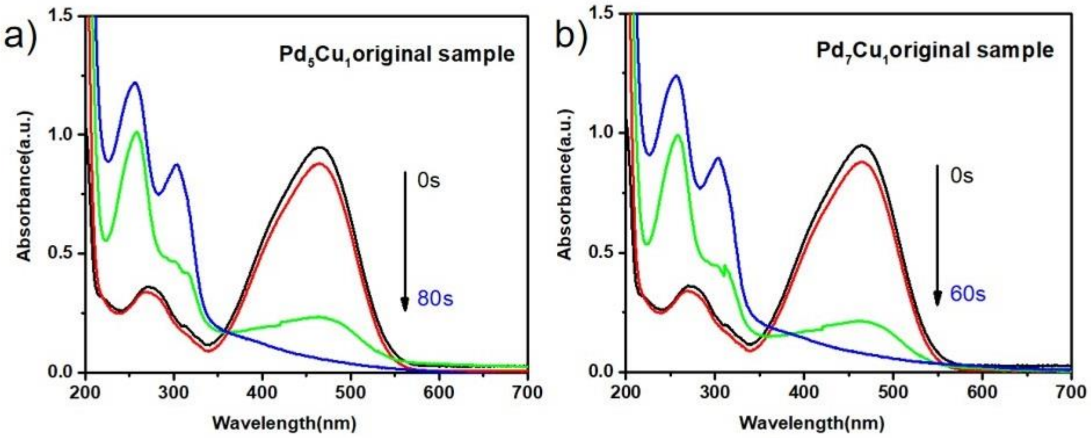

Figure. S10. UV-*vis* absorbance spectra of degradation of 0.06 mM MO by $NaBH_4$ in the presence of 100 μL of Pd_5Cu_1 and Pd_7Cu_1 alloy NPs.

Supporting Information

Table S4: Summary of Comparative Reduction Time (t), Apparent Rate Constant (k_{app}), and Activity Parameter (K) for Different MTANP Samples. The concentration of substrate MO was controlled at 0.06 mM, and the content of $NaBH_4$ was controlled at 100 µL (0.1 mM). The effect of different catalysts on reaction rates can be observed in the table below.

Sample name	Reduction time(s)	Apparent rate constant (K_{app}'$10^{-3}s^{-1}$)
Pd_5Cu_1	80	40.1
Pd_7Cu_1	60	53.5
Pd_5Cu_1(10MPa,3000W)	30	107
Pd_5Cu_1(10MPa,4000W)	18	178.3
Pd_7Cu_1(10MPa,3000W)	20	160.5
Pd_7Cu_1(10MPa,4000W)	9	356.7

Supporting Information

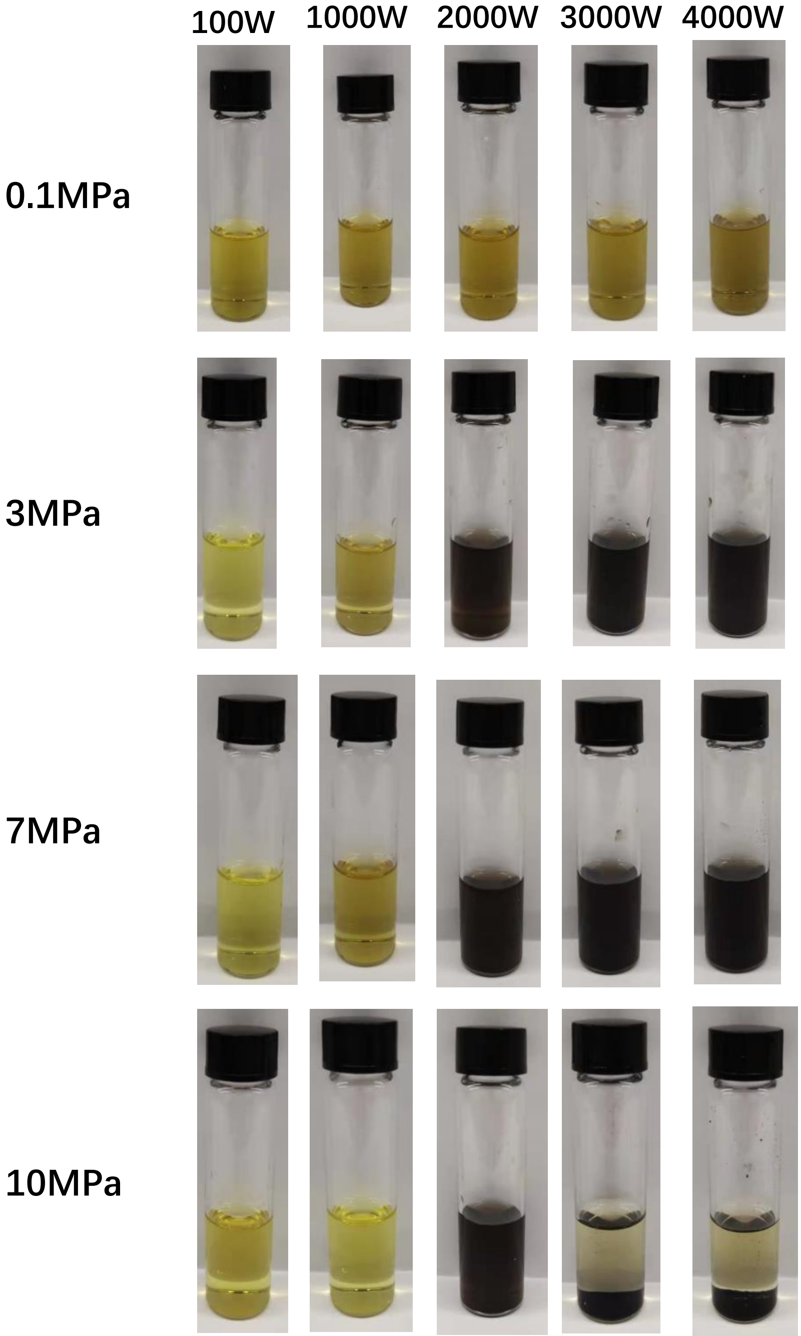

Figure. S11. Solution states of Pd_5Cu_1 alloy NPs affected by different ultrasonic parameters.

Supporting Information

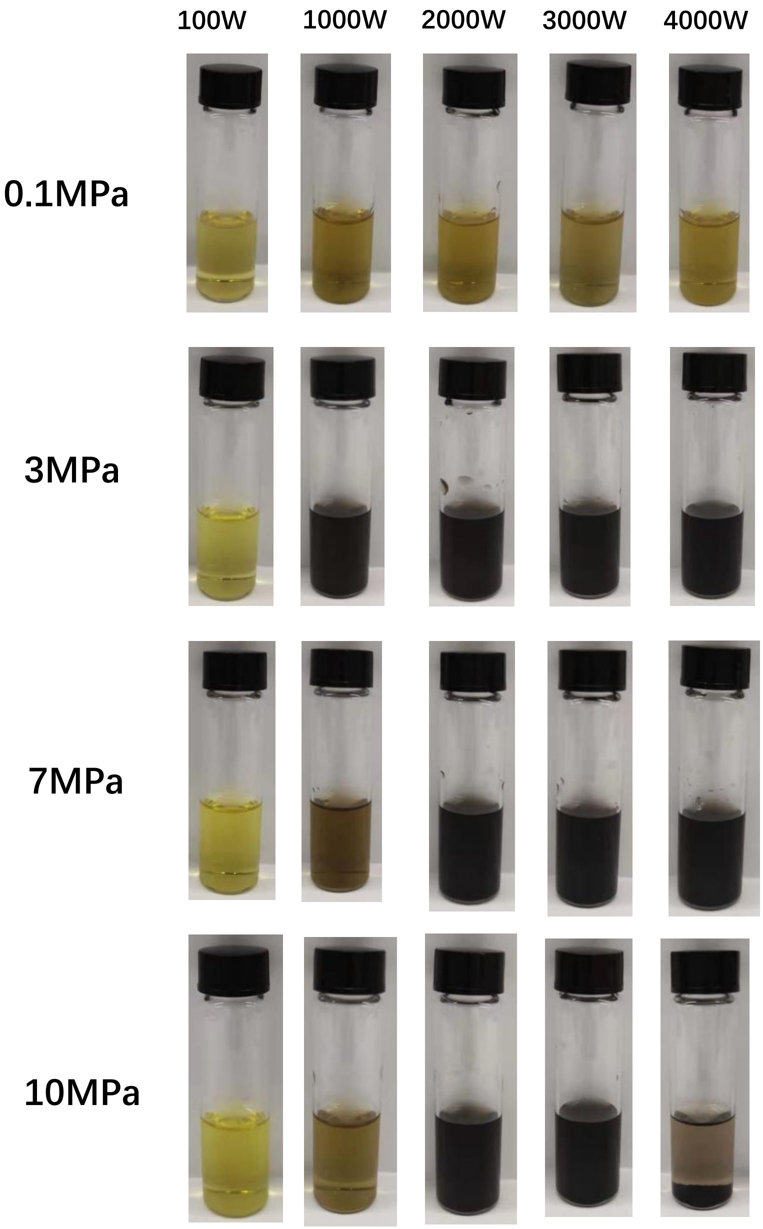

Figure. S12. Solution states of Pd_7Cu_1 alloy NPs affected by different ultrasonic parameters.
